# Systems thinking to understand the complexity of antimicrobial resistance across One Health: A systematic review of current approaches

**DOI:** 10.1016/j.onehlt.2025.101081

**Published:** 2025-05-22

**Authors:** Yen Pham, Teresa M. Wozniak

**Affiliations:** a Australian e-Health Research Centre, CSIRO, Brisbane, Qld 4029, Australia; b James Cook University, Qld, Australia

**Keywords:** Antimicrobial resistance, Antibiotic resistance, Systems thinking, Systems maps, System dynamics, Causal loop diagrams, Stock and flow diagrams, One health

## Abstract

Antimicrobial resistance (AMR) is a significant global threat that affects not only human health but also the health of animals and the environment. The evolution and spread of AMR are driven by a complex set of interconnected factors across all domains of One Health. Effectively addressing this challenge requires systems approaches and collaborative efforts across multiple sectors. We conducted a systematic quantitative literature review to explore the application of systems thinking approaches in examining the factors driving AMR and their interactions across human, animal and environmental systems. Based on 21 selected studies, we developed a causal loop diagram to illustrate key contributing factors and their interrelationships influencing AMR and highlight the need for interventions that extend beyond antibiotic use. Our findings emphasise that reducing antibiotic use in a single system is insufficient to curb AMR. Instead, sustained reductions require a multifaceted approach, including stronger regulations, increased awareness of appropriate antibiotic prescribing and use, non-antimicrobial measures such as vaccination and infection control, and improved waste and wastewater treatment practices. Despite progress in understanding AMR, critical gaps remain in assessing how social determinants, human activities, and environmental factors contribute to its evolution and spread. Systems thinking has proven valuable in fostering a shared understanding of AMR, facilitating collaborative decision-making, and informing evidence-based policies. Engaging stakeholders in co-designing and evaluating interventions will be essential to advancing global efforts to mitigate AMR and achieve long-term, sustainable solutions.

## Highlights

• Systems thinking examines interactions among drivers of AMR across One Health.
• Feedback loops among human, animal and environmental systems are identified.
• A causal loop diagram highlights interventions beyond antibiotic use.
• Systems thinking fosters a shared understanding and responsibility for AMR.
• Socio-economic dynamics should be integrated into AMR strategies.

## Graphical abstract

**Figure:**
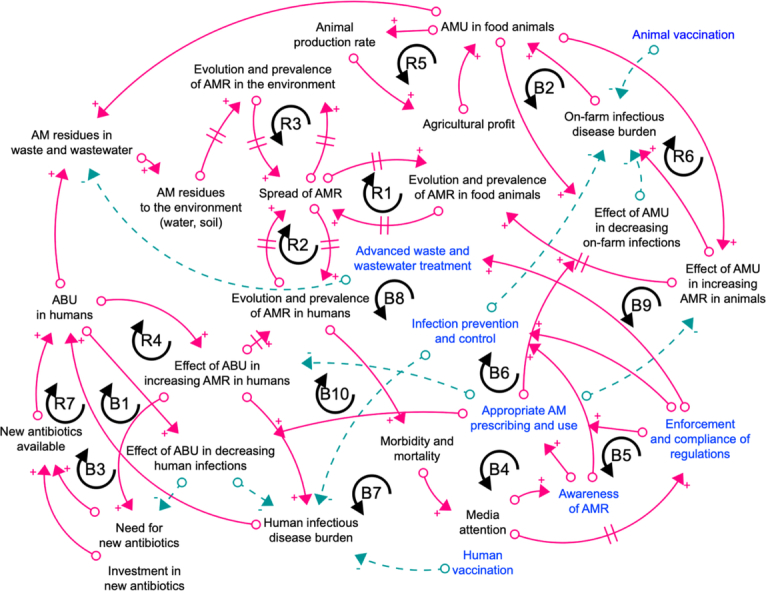

## 1 Introduction

Antimicrobial resistance (AMR) is recognised as a global public health threat, demanding urgent action [48]. In 2019, bacterial AMR was responsible for an estimated 4.95 million deaths, including 1.27 million directly attributable to AMR [31]. The burden varies significantly across regions, with Australasia experiencing the lowest AMR-related attributable deaths (6.5 per 100,000 population), while western sub-Saharan Africa faces the highest burden (27.3 per 100,000 population) [31]. Projections indicate that by 2050, there could be 1.91 million deaths directly attributable to AMR and 8.22 million deaths associated with AMR annually [32]. While the consequences of AMR are most apparent within clinical settings, the evolution and spread of resistant pathogens extend beyond human health [5,11]. Antimicrobials consumed by humans or animals—or resistant pathogens residing in these hosts—are excreted as waste, and if not adequately treated, resistant genes and antimicrobial residues are released into the environment [25]. Persistent anthropogenic activities, such as introducing heavy metals [18], using sewage for irrigation and applying livestock manure [41] exert selective pressure on microbial communities. This can promote the horizontal transfer of resistant genes between bacteria in the environment, increasing the reservoir of these genes and the risk of their transfer to humans and animals [50].

AMR is a complex system challenge driven by the interaction of multiple factors across human, animal and environment health, also known as One Health sectors. The dynamic complexity of AMR arises from interrelated processes, feedback mechanisms and emerging properties within the One Health ecosystem [5]—characteristics of a complex system [35,44]. Importantly, the causal relationships between factors driving AMR are not straightforward and can evolve in unexpected ways. For example, interventions in one area, such as restrictions on antimicrobial use in agriculture, can lead to unintended consequences in other systems [30], highlighting the necessity for a holistic approach.

Systems thinking is a framework that helps us understand how different variables within a complex system are interconnected [43]. It provides valuable methods and tools for analysing these interrelationships and creating long-lasting interventions for complex problems [28]. The systems thinking approach is well suited to addressing the intersecting factors contributing to AMR across all One Health domains [11,26] and identifying leverage points that enable sustainable change in controlling the AMR threat [4,5].

Limited evidence exists regarding the application of systems thinking to AMR, despite its use in various sectors such as economics [10], climate policy [12], agriculture [36] and public health [40]. Whilst there is agreement that AMR exists within One Health systems, there remains limited understanding of the dynamic relationships and feedback mechanisms driving AMR. This knowledge gap hinders our ability to effectively reduce the threat of AMR.

This systematic quantitative review aims to address this gap by answering the following question: “What systems thinking-based approaches are applied to examine the interrelationships between factors influencing the evolution and spread of AMR across One Health systems?”. Our focus is on systems mapping and system dynamics, including tools such as causal loop diagrams (CLD) and stock and flow diagrams (SFD) to capture and/or simulate the hypothesised causal relationships or feedback mechanisms driving AMR. Both systems mapping and CLDs are valuable for visualising complex systems by conceptualising relationships between system components. Systems mapping identifies the connections, while CLDs reveal how those connections influence each other through causal links and feedback loops, enabling a deeper understanding of system behavior [49]. Stock and flow diagrams (SFDs) are quantitative tools commonly used to design and analyse policy scenarios in simulated, dynamic environments by incorporating feedback to capture the non-linear dynamics of complex systems [44].

## 2 Materials and methods

Following the methods presented in Pickering and Byrne [37], we conducted a systematic quantitative review of the academic literature on the application of systems thinking-based approaches for AMR. The review process followed the 10-step process of knowledge creation detailed in Pickering et al. [38] to collect and analyse relevant data, including 1) Define the topic; 2) Formulate research questions; 3) Identify keywords; 4) Identify and search databases; 5) Read and assess publications; 6) Structure the database; 7) Enter first 10 % papers; 8) Test and revise categories; 9) Enter bulk of papers; and 10) Produce and review summary tables.

### 2.1 Selection criteria and eligibility

Our search focussed on original research papers published in peer-reviewed English-language academic journals until March 2024. Publications such as review articles, position, commentary or protocol papers, book chapters, reports and conference proceedings were not included.

Our inclusion criteria were that research had to apply systems thinking approaches to (1) understand factors influencing AMR and their impact in human or non-human (i.e. animal or environmental) settings; or (2) propose or evaluate interventions to mitigate AMR infections or AMR-related mortalities.

Research might not necessarily analyse why certain factors influence the emergence and spread of AMR but imply this possibility; for example, overuse or misuse of antibiotics attributed to inappropriate prescribing, or antibiotic concentration in natural water bodies resulting from antibiotic use in agricultural production. Such studies are included in this review.

### 2.2 Information sources and search strategy

Applying a set of search terms, we surveyed the literature in three scholarly electronic databases, including Scopus, Web of Science, and Medline to identify relevant journal articles. The search strings used were combinations of terms associated with systems thinking and AMR (Table 1). Note that similar search strings were also applied separately for anti-fungi, anti-virus and anti-parasite; however, all results returned are irrelevant.

**Table 1:** The search strategy for the review.

Database	Search strings
Scopus	TITLE-ABS-KEY ((“system* thinking” OR “system* dynamic*” OR “causal loop” OR “system* science” OR “system* theor*” OR “dynamic* system*” OR “system* map*” OR “system* approach*”) AND ((anti*microb* OR anti*b* OR multi*drug* OR microb* OR drug* OR extensive*drug*) W/5 resis*) OR superbug*)
Web of Science	TS = (“system* thinking” OR “system* dynamic*” OR “causal loop” OR “system* science” OR “system* theor*” OR “dynamic* system*” OR “system* map*” OR “system* approach*”) AND TS = (((anti*b* OR anti*microb* OR microb* OR drug* OR multi*drug* OR “extensive*drug”) NEAR/5 resis*) OR superbug*)
Medline	TS = (“system* thinking” OR “system* dynamic*” OR “causal loop” OR “system* science” OR “system* theor*” OR “dynamic* system*” OR “system* map*” OR “system* approach*”) AND TS = (((anti*b* OR anti*microb* OR microb* OR drug* OR multi*drug* OR “extensive*drug”) NEAR/5 resis*) OR superbug*)

### 2.3 Screening and selection

Following the search, all identified records were collated and uploaded into Endnote (Version 20). Titles and abstracts were screened for assessment against the eligibility criteria for the review. Potentially relevant articles were retrieved as full texts and assessed in detail against the inclusion criteria. Reasons for exclusion at the full-text screening stage were recorded. The results of the full search and the article selection process are presented in a Preferred Reporting Items for Systematic Reviews and Meta-Analyses (PRISMA) flow diagram.

### 2.4 Data extraction and synthesis

Data were extracted into a customised database, including information on geographic focus, objectives and the core problem to be addressed within each study. The main themes of One Health focus areas associated with AMR and the main drivers of AMR detailed in the literature were entered into the database to identify their patterns and gaps and to inform future research. Details of systems thinking approaches such as the type of tools and software used, main methods to support such tools, the type and source of data and methods to validate results from using systems thinking tools as well as analysis of feedback loops, leverage points, simulation, prediction, and intervention scenarios were also recorded for the review.

## 3 Results

**Fig. 1:**
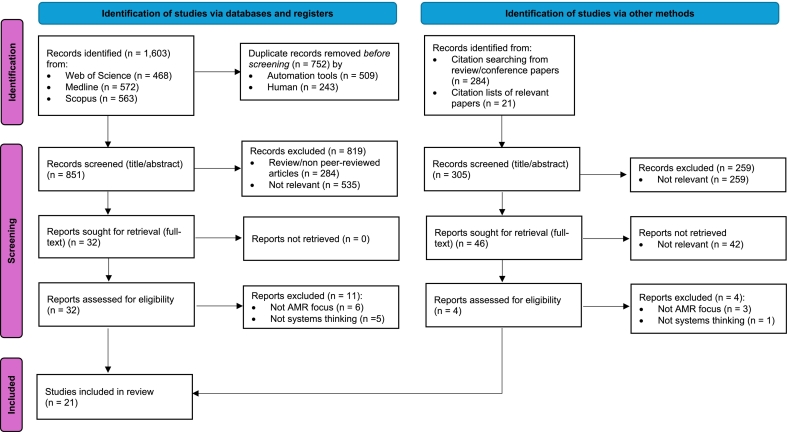
PRISMA flow diagram of the screening process for study selection (adapted from [34]), n = number of records.

Database searches retrieved 1603 records, of which 752 duplicates were automatically and manually removed using Endnote (Fig. 1). There were 851 records screened based on their titles and abstracts; of these, full texts of 32 articles were reviewed with 21 articles meeting the inclusion criteria. The reasons for excluding 11 articles are neither sufficiently focused on AMR nor the use of systems thinking approaches. Checking reference lists of the 284 review and conference papers and the 21 relevant articles resulted in no additional articles to be included. A total of 21 relevant papers were selected to be fully examined in this review.

### 3.1 Characteristics of selected studies

The characteristics of the 21 selected articles are outlined in Table 2. The majority of articles analysed (*n* = 15, 71 %) were published within the last five years (2019–2023), suggesting an increasing interest in systems thinking approaches which was also identified in published health research over the past 15 years [47]. The geographical scope of articles included regional or multi-national (*n* = 6), national (*n* = 10) and sub-national (*n* = 5). Articles were predominantly from European countries (*n* = 12), followed by African countries (*n* = 7) and Asian countries (*n* = 2).

**Table 2:** Characteristics of selected studies.

Author (published year)	Geographic focus	Research objective	Research problem	One Health domain (focus area)	Systems thinking tool(s) (software used)	Method(s) to support systems thinking tools	Type of data (source)	Method(s) to validate results
Aboah et al. [1]	Senegal	Visualise impact of AMR in poultry production and identify interventions	AMR driven by AMU in poultry production	Food-producing animal (poultry)	CLD (Vensim)	Workshop	Qualitative (Participant input)	Workshop participants
Adamu et al. [2]	Kano state, Nigeria	Investigate factors driving non-prescription sales of antibiotics	ABR driven by non-prescription sale/irrational use of antibiotics	Human	CLD (Vensim)	Semi-structured questionnaire/ cross-sectional surveys	Qualitative (Participant input)	NS
Atun et al. [6]	Samara Oblast, Russia	Quantify impact of a program on MDRTB control under HIV epidemic	Deaths from tuberculosis including MDRTB	Human	SFD	Previously published models	Quantitative (Statistics and published estimates)	Historical data
Atun et al. [8]	Estonia	Quantify impact of harm-reduction program and MDRTB control on cumulative HIV/AIDS, tuberculosis and HIV-associated tuberculosis deaths	Deaths from tuberculosis including MDRTB	Human	SFD	Previously built model in Atun et al. [6]	Quantitative (Literature, explicit assumptions and surveillance data)	Historical data
Atun et al. [7]	Russia	Estimate impact of HAART and cure rates for MDRTB under HIV epidemics	Deaths from tuberculosis including MDRTB	Human	SFD	Previously built model in Atun et al. [6] and Atun et al. [8]	Quantitative (Literature, explicit assumptions and statistics)	Historical data
Brunton et al. [9]	Mekong, Vietnam	Analyse drivers of ABR and human exposure to ABR in aquaculture systems	Exposure to ABR in aquaculture and the wider environment	All areas (Aquaculture)	Systems map (Lucidchart)	Workshops	Qualitative (Stakeholder input)	Workshop participants, content experts and literature
Cousins et al. [13]	Sweden	Quantify factors driving AMR and the strength of their relationships	Lack of empirical data for quantitative modelling of AMR	All areas	CLD	Workshops	Qualitative (Stakeholder input)	NS
Cox Jr. and Ricci [14]	EU and USA	Analyses consequences of well-intended policies for promoting food safety	ABR driven by ABU in animal	Animal, Human	SFD	Literature review to identify causal relations between model variables	Quantitative (Published data)	NS
Desbois et al. [15]	Nile Delta, Egypt	Analyse interventions to reduce ABU in tilapia farming	Exposure to ABR in aquaculture	All areas (Aquaculture)	Systems map (Lucidchart)	Workshop	Qualitative (Participant input)	Workshop participants
Frolova et al. [16]	Kaban lakes, Russia	Investigate antibiotic concentration in a lake system	Antibiotic residues in water bodies driven by antibiotic substances from medical and agricultural use	All areas (environment)	CLD and SFD (Stella)	Previously built model in Sverdrup et al. [45]	Quantitative (Literature and published data)	NS
Glover et al. [17]	UK	Investigate interactions between antibiotic prescribing interventions and the wider health system	Complex local system of antibiotic prescribing and interventions that might impact AMR	Human	Systems map (Kumu)	Interviews	Qualitative (Interviewee input)	Literature
Homer et al. [19]	USA, Spain, South Africa and Hungary	Investigate ABU-related interventions to avoid a ABR epidemic	High rates of illness and deaths due to ABR driven by inappropriate ABU	Human	SFD	Expert meeting and literature review	Mixed (expert input, literature and published data)	Historical data
Kianmehr et al. [20]	USA	Simulate antibiotic prescribing behavior and evaluate impact of relevant interactions	ABR in human driven by inappropriate antibiotic prescribing	Human	SFD	Literature review to identify causal links for the model	Quantitative (empirical data)	Literature and input from experts
Kiekens et al. [21]	Tanzania	Investigate interactions between HIV drug resistance-related factors as a complex adaptive system	Threats of HIV drug resistance to antiretroviral therapy	Human	CLD (Kumu)	Interviews	Qualitative (interviewee input)	Workshop participants
Kiekens et al. [22]	Sub-Saharan Africa	Investigate factors influencing HIV drug resistance	Threats of HIV drug resistance to antiretroviral therapy	Human	CLD (Kumu)	Interviews	Qualitative (interviewee input)	NS
Lambraki et al. [23]	Europe	Investigate factors influencing AMR in the European food system and leverage points for interventions	AMR in the food system driven by AMU and other complex factors	All areas (food system)	CLD (Vensim)	Workshop	Qualitative (participant input)	Workshop participants
Lambraki et al. [24]	Southeast Asia	Investigate factors influencing AMR in the Southeast Asian food system and leverage points for interventions	AMR in the food system driven by AMU and other complex factors	All areas (food system)	CLD (Vensim)	Interviews and workshops	Qualitative (participant input)	Workshop participants
Lebcir et al. [27]	Russia	Explore the dynamic transmission of tuberculosis, MDRTB and HIV and the impact of different interventions	Deaths from tuberculosis including MDRTB	Human	CLD and SFD	Literature review, expert discussions and interviews	Mixed (Published estimates for quantitative data)	Expert input, historical data
Matthiessen et al. [29]	No focus	Investigate interconnections between human, animal and environmental components associated with AMR in a circular systems map model	AMR in all One Health spectrum driven by AMU	All areas	Systems map (Kumu)	Literature review	Qualitative (literature)	NS
Sverdrup et al. [45]	Volga River, Russia	Quantify antibiotic concentration in a river system	Antibiotic residues in waterways as a result of antibiotic substances from medical and agricultural use	All areas (environment)	CLD and SFD (Stella)	Literature review	Quantitative (Literature and assumptions)	None
Zhu et al. [51]	UK	Investigate impact of decision-making for antibiotic prescribing in hospitals	AMR in human driven by inappropriate antibiotic prescribing	Human	CLD and SFD (iThink)	Literature review	Mixed (empirical data from interviews, surveys and hospital data)	Expert discussions

Abbreviations: ABR: Antibiotic resistance; ABU: Antibiotic use; AIDS: Acquired immunodeficiency syndrome; AMR: Antimicrobial resistance; AMU: Antimicrobial use; CLD: Causal loop diagram; HAART: Highly active antiretroviral therapy; HIV: Human immunodeficiency virus; MDRTB: Multidrug-resistant tuberculosis; NS: Not specified; SFD: Stock and flow diagram

Eleven articles focused exclusively on human health (52 %), three on animal health, two on food systems and two on environmental systems. Less than half of the articles considered the impact of AMR across all One Health domains (*n* = 8). For example, the interactions of AMR drivers between human and animal systems. Articles reported on resistance in bacteria (*n* = 10), resistance in viruses (*n* = 2) and AMR (*n* = 7).

Among the eleven human health articles, five studies applied systems thinking to analyse antibiotic prescribing and use, four focused on multidrug-resistant tuberculosis and two studies assessed resistance to Human Immunodeficiency Virus.

Among the non-human health articles, one study focused on antibiotic use and resistance in food-producing animals (poultry); two studies investigated antibiotic resistance in aquaculture systems; two studies assessed factors influencing AMR in food systems; and two studies measured antibiotic concentration in water bodies as a result of antibiotic use in medical and agricultural activities.

There were two articles focusing on all areas of One Health and one article across the human and animal health space, investigating zoonotic diseases and the use of antibiotics to treat infections in the human and animal systems.

The systems thinking approaches used in the articles were both qualitative (*n* = 11), quantitative (*n* = 6) and mixed methods (*n* = 4). Qualitative methods included systems mapping developed from literature reviews and stakeholder workshops (*n* = 4) and CLDs developed based on stakeholder consultation through interviews, surveys or workshops (*n* = 7).Quantitative methods used SFD formulated based on previously built models, literature, expert discussions and assumptions (*n* = 6). The four mixed methods studies applied both CLD and SFD using input from literature, expert discussions, interviews and previously published models.

Five software packages were used, including Vensim (*n* = 4) and Kumu (*n* = 3) for developing CLD, Kumu (*n* = 2) and Lucidchart (*n* = 2) for systems mapping, and Stella (*n* = 2) and iThink (*n* = 1) for SFD.

Regarding methods for validating results from using systems thinking tools, two studies used literature and six studies used input from stakeholder workshops and expert discussions to validate CLD or systems maps, five studies used historical data and three studies used input from experts to validate results of system dynamics models.

To understand the process of developing systems maps and models, details of systems thinking approaches applied were extracted from the selected studies and captured in Table 3. Eleven studies applied a participatory approach with stakeholder involvement to develop systems maps, CLDs or SFDs, of which seven studies indicated the application of a group model-building exercise. Such approaches maximise the participation of all relevant stakeholders in all stages of the research and decision-making process, incorporating diverse views and leading to ‘*a sense of collective responsibility towards the problem… and shared ownership towards solutions*’ [15].

**Table 3:** Details of systems thinking approaches and tools applied to support their use in selected studies.

Details of systems thinking approaches and support tools	Number of studies	References
Group model building (workshops, expert discussions)	7	[1,9,15,19,24,23,27]
Qualitative analysis of leverages/interventions	7	[1,9,15,17,24,23,29]
Prediction analysis	7	[6,7,8,16,19,45,51]
Quantitative analysis of intervention scenarios	7	[6,7,8,19,27,45,51]
Validation of simulation models	6	[7,8,19,20,27,51]
Combination with other technique • Statistical modelling • Network analysis	1 1	[2] [1]

Seven studies suggested potential leverage points or places for interventions to control AMR or minimise its impacts. Among ten studies developing simulation models from SFDs, seven provided prediction analysis about AMR-related trends. Seven studies quantitatively analysed various intervention scenarios associated with antimicrobial usage- or AMR-induced outcomes compared to business-as-usual scenarios. Validating results of simulation models were performed in six studies and combining systems thinking with other approaches was indicated in two studies, including statistical model and network analysis.

### 3.2 Intended use of systems thinking approaches

There were three main ways that systems thinking approaches were used in selected articles (Table 4). The most common use of systems thinking was to facilitate collaborative actions on AMR by engaging stakeholders involved in and affected by decision-making processes. This included promoting a shared understanding of AMR (*n* = 5), and co-identifying leverage points and potential intervention strategies (*n* = 4) and assessing the impact of policy and management decisions (*n* = 2). Another key application was supporting decision-making, particularly through the design of intervention scenarios to provide policy implications and recommendations while comparing the outcomes of various policy scenarios (*n* = 7). Lastly, systems thinking was used to inform future research, including identifying knowledge gaps and foundational elements for formulating intervention strategies (*n* = 5) and promoting whole systems thinking and approaches in AMR research (*n* = 3).

**Table 4:** Intended use of systems thinking approaches in selected studies.

Intended use	Number of studies	Examples including references
*Facilitate collective views through stakeholder engagement and support decision-making*		
Promote a shared understanding of the complexity of the problem	5	Provide a platform for stakeholders to: - Understand the complex nature of AMR in poultry production [1] - Characterise the interconnected factors related to non-prescription antibiotic sales [2] - Foster interactions between subject experts and combine diverse insights into a framework (i.e. maps of elements associated with ABU/ABR in two aquaculture systems) [9] - Integrate perspectives from various stakeholders to generate or expand understanding of AMR evolution and spread, and its impact on food systems, including actions that may influence AMR ([24], [23])
Co-conceptualise leverage points/interventions	4	Provide stakeholders with opportunities to: - Discuss antibiotic alternatives and potential interventions to improve fish health [15] - Identify and prioritise intervention pathways to enhance agricultural profitability and reduce AMR [1] - Identify leverage points for targeted interventions to reduce AMR in food systems ([24], [23])
Co-identify unintended consequences of policy/management decisions beyond their immediate impact	2	Permit an understanding of: - The overuse of antimicrobials–an economic decision made by producers to improve production efficiency and profit–as a driver of AMR [1] - The unintended consequences associated with AMR stemming from actions/decisions influenced by food insecurity, poverty and environmental conditions [24]
Design intervention scenarios that provide policy implications and recommendations	7	System dynamics models were developed to: - Estimate the impact of strategies aimed at reducing the tuberculosis-related deaths and inform future communicable disease policies [[6], [7], [8],^27^] - Evaluate well-intentioned food safety policies associated with animal antibiotic use and their impact on animal and human health [14] - Assess various scenarios to reduce antibiotic contamination in waterways through agricultural antibiotic use and sewage treatment [45] - Simulate intervention scenarios for optimal antibiotic prescribing in hospitals, including staff compliance with guidelines and rapid point-of-care testing [51]
*Inform future research*		
Provide insights into the system complexity, including knowledge gaps and foundations for formulating and evaluating quantifiable intervention strategies	5	Systems thinking approaches help to: - Identify knowledge gaps, such as the lack of information on ABU at various points within aquaculture systems and the integration between aquaculture production and other food systems [9] - Highlight the need to examine factors beyond ABU that contribute to AMR in poultry production [1] - Focus on interventions that incorporate ABU and ABR training tailored to the context of non-prescription antibiotic sales [2] - Provide insights to assess interventions that reflect the dynamics of local prescribing systems [17] - Offer a simulation framework to design and evaluate future interventions for optimal antibiotic prescribing [20]
Encourage non-linear and whole-systems thinking and approaches in future research on AMR	3	Mapping the system in which AMR evolves and spreads (e.g., through antibiotic flow) and identifying leverage points is more effective with non-linear thinking [9] Adopting a whole-system lens to examine complex antibiotic prescribing systems can lead to sustainable interventions and prevent unintended consequences arising from temporary solutions (e.g., the impact of austerity and other non-AMR policies on AMR) [17] Encouraging scientists to move away from linear thinking and incorporate systems thinking in future research can support the development of more effective interventions [29]

Abbreviations: ABR: Antibiotic resistance; ABU: Antibiotic use; AMR: Antimicrobial resistance

### 3.3 Risk factors influencing AMR evolution and spread

**Fig. 2:**
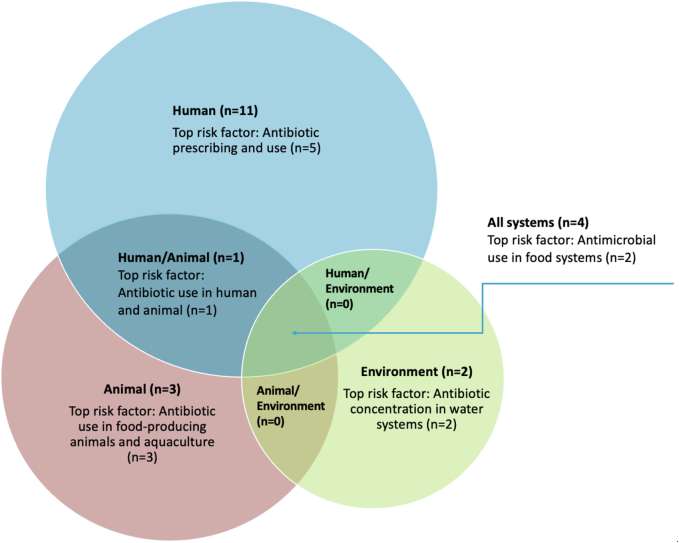
Main risk factors influencing AMR in One Health systems identified in selected studies (n = number of studies).

Fig. 2 illustrates the overlap of potential top risk factors identified in the selected studies which may be attributed to influencing AMR evolution and spread across One Health systems. Five articles [2,17,19,20,51] reported inappropriate prescribing and/or consumption of antibiotics as the main risk factor for AMR in human health. Three studies [1,9,15] reported inappropriate antimicrobial use in food-producing animals and aquaculture leading to human exposure to AMR both from animal and environmental reservoirs. Two studies [16,45] reported the concentration of antibiotics in river systems as a risk factor influencing AMR emergence and spread in the environment. Antibiotic concentration in such systems was the result of antibiotic substances from medical (human) and agricultural (animal) uses. One study [14] reported antibiotic use in both human healthcare and animal production leading to the emergence and transmission of AMR between these two systems. Two studies [23,24] identified the overlap of all One Health domains while reporting the risk factor of AMR in food systems.

### 3.4 A causal loop diagram of factors influencing AMR

**Fig. 3:**
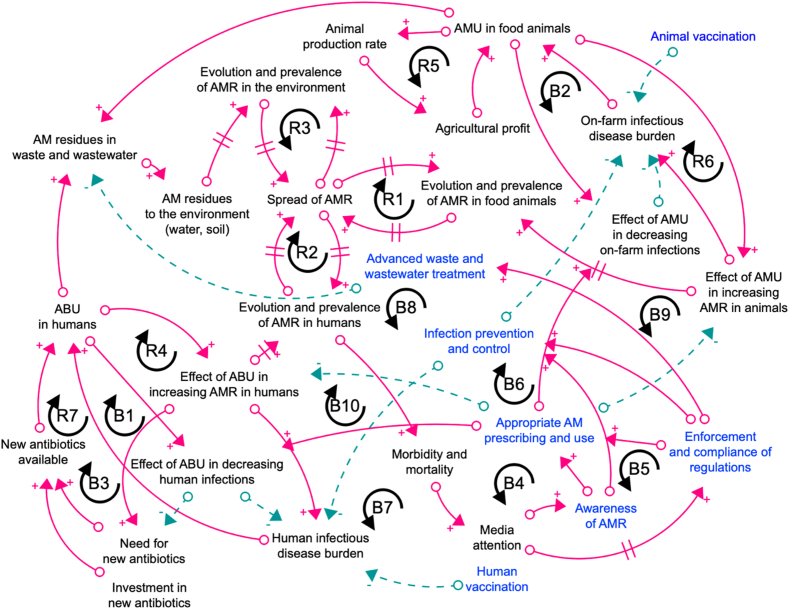
A causal loop diagram of the interactions between key factors influencing AMR based on selected studies. : Positive relationship; : Negative relationship; //: Delay; : Potential leverage points for targeted interventions to address AMR. Abbreviations: AM: Antimicrobials; ABU: Antibiotic use; AMR: Antimicrobial resistance; AMU: Antimicrobial use; R: Reinforcing loop; B: Balancing loop

Using the selected articles that identified risk factors influencing AMR evolution and spread and their interrelationships across One Health, we developed a CLD using Stella Architect (version 3.8) (Fig. 3) to represent the interactions between risk factors of AMR. However, only connections between key factors influencing AMR that constitute feedback loops were captured in our CLD (refer to Table A.1 in the Appendix for a detailed description of each relationship included in the CLD).

A CLD includes feedback processes within a system that represent dynamic hypotheses about the causes of the problem [44]. It comprises reinforcing (R) and balancing (B) loops to characterise mechanisms influencing the dynamics of the system. Reinforcing loops portray growing or declining actions while balancing loops counteract or self-regulate to seek equilibrium or a specified target in the system [28]. Feedback loops are formulated based on the connections (illustrated by arrows) between variables or factors within a system. A positive (+) relationship means two factors change in the same direction. On the contrary, a negative (−) relationship describes the opposite direction between two elements. CLDs may involve ‘delay’ (//) which represents the time lag between a cause (or action, input) and its effects and is often the trade-offs among short- and long-term impacts of policies that might cause undesirable consequences. For example, reducing antibiotic usage does not lead to an immediate decrease in resistance due to system delays, and there is no evidence to date of resistance reversal [39].

Details of relationships in sub-systems (i.e. human, animal or the environment) not fully captured as the intention of developing this CLD was to represent how AMR fits within the broad context of One Health. For example, the dynamics influencing antibiotic prescribing and use can be further explored from both clinical and community settings. Depending on the system boundary defined by the investigators or stakeholders, the level of details of the system in which the problem occurred can be explored. In this review, only interactions, mainly between direct factors influencing AMR, that can be identified from the selected studies were included.

Overall, we identified seven reinforcing and 34 balancing loops in the CLD that captured the main drivers of AMR and their interdependencies across One Health domains. The structure of each feedback loop is specified in Table A.2 in the Appendix. For example, as illustrated in balancing loop B1, ABU in humans aims to reduce infections, meaning that an increase in ABU *(‘ABU in humans’)* is expected to enhance its effect on decreasing infections *(‘Effect of ABU in decreasing human infections’)*, thereby reducing the infectious disease burden *(‘Human infectious disease burden’)*, and ultimately leading to a decrease in ABU *(‘ABU in humans’)*. However, reinforcing loop R4 explains why loop B1 may not always dominate in the system. Specifically, an increase in ABU *(‘ABU in humans’)* is also likely to amplify its effect on increasing AMR in humans *(‘Effect of ABU in increasing AMR in humans’)*, which in turn raises the infectious disease burden *(‘Human infectious disease burden’)*, and ultimately drives further increases in ABU *(‘ABU in humans’)*.

## 4 Discussion

This review highlights how systems thinking can be applied to identify key risk factors and their interactions that influence the evolution and spread of AMR across One Health systems. By understanding these interconnected systems, systems thinking helps identify leverage points for targeted interventions, enabling long-term and sustainable change. This approach promotes collaboration between stakeholders, fostering a shared understanding and coordinated actions necessary to address the complex issue of AMR.

In this review, we present a CLD developed from factors driving AMR identified in the selected studies and their interrelationships across One Health systems. The CLD highlights antimicrobial use (primarily antibiotics) in both human and animal systems, and antibiotic concentration in the environment as the main drivers of AMR. While antibiotics are essential in reducing infections caused by infectious disease burden (loop B1), their overuse potentially contribute to AMR (loop R4). However, decreasing usage does not necessarily result in an immediate reduction in resistance due to system delays, as reflected in current literature. Research indicates that reducing antimicrobial usage alone is not sufficient to mitigate the AMR threat [39].

Key leverage points or potential areas for targeted interventions to reduce AMR identified from the selected studies were captured in our CLD. The first leverage point is to ensure appropriate prescribing and antibiotic use in both human and animal health. Achieving this will require increasing AMR awareness among healthcare practitioners to ensure compliance with relevant regulations and raising public awareness to ensure adherence to prescribed treatments. The second leverage point is vaccination and infection prevention and control measures, which help prevent or reduce the incidence of infections and consequently the need for antibiotics. The third leverage point focuses on advanced waste and wastewater treatment practices, which help limit environmental contamination with antibiotic residues, thereby reducing the spread of AMR from the environment back into human and animal systems. It is argued that no single leverage point is a silver bullet that can address AMR on its own, as each interacts with other factors within the CLD, and these multidimensional interactions collectively drive AMR. While the selected studies identified numerous leverage points, not all of them were captured in the CLD, as some were context-specific or involve additional feedback loops that were beyond the scope of this review.

Half of the studies in human health focused on inappropriate antibiotic prescribing and use, yet insights into the underlying dynamics driving this behavior were limited. The social determinants of health are considered key drivers of AMR evolution and spread. For example, Schmiege et al. [42] reported that factors driving antibiotic use within community included, but were not limited to, education, employment, income and antibiotic regulations. Additionally, poverty, living conditions, and access to basic amenities and healthcare services were reported to influence AMR [33]. Only two systems thinking studies emphasised the importance of incorporating socio-economic dynamics in implementing AMR interventions, highlighting the risks of focusing solely on downstream factors without adequately considering local contexts such as poverty [17] or rapidly changing environments in which AMR occurs [29]. Neglecting these may result in the development of short-term solutions that likely provide only temporary benefits. Such solutions, often referred to as ‘quick fixes’ in systems thinking, may lead to unintended consequences that undermine the effectiveness of interventions [28]. For example, the use of new antibiotics may temporarily reduce the infectious disease burden in the short term (loop B3); however, inappropriate use may ultimately worsen the burden over time due to AMR (loop R4 and R7).

This review highlights that only a limited number of studies in the animal and environmental sectors have applied systems thinking to understand the evolution and spread of AMR. Notably, no studies assessed AMR in companion animals, wildlife or plant health, reflecting trends observed in other AMR research approaches within these sectors [3,11,46]. In environmental health, systems thinking was applied in only two studies, both of which examined antibiotic residue concentrations in river systems resulting from medical and agricultural use, while no studies explored AMR in soil or plants. Future research should focus on the interdependencies between factors influencing AMR (and associated genes and residues) within animal and environmental systems, as well as their interactions with human activities. This will be essential for identifying key leverage points and designing interventions that can drive system-wide change to minimise AMR.

From the selected studies, those that developed simulation models primarily focused on human health. This may reflect data constraints in the environmental and animal sectors, as well as limited understanding of the feedback mechanisms between AMR risk factors across these systems. While reliance on model assumptions due to data gaps may increase uncertainty, the primary goal of most systems modelling approaches is to improve understanding of system behavior under different scenarios rather than to produce precise predictions [44].

The CLD produced from the selected studies is not an exhaustive conceptual model and may have excluded certain risk factors, leverage points and their potential interactions. Incorporating all identified factors from each study is not practical; for example, Lambraki et al. [23] identified 91 factors and 331 connections in a CLD addressing AMR within a European food system. The primary objective of constructing the CLD was to capture the feedback mechanisms between key AMR-related factors across One Health systems without being confined to a specific health setting or geographical area. This model can serve as a foundation for future research applying systems thinking approaches to address AMR in one or more One Health systems.

## 5 Conclusions

This review highlights the benefits of using systems approaches to tackle complex challenges like AMR. It identifies key risk factors, including antibiotic use in human and animal health and environmental contamination with antibiotic residues, as the main drivers of AMR across all One Health sectors. By developing a CLD, we demonstrate that reducing antibiotic use in any one system alone will be insufficient to control AMR. Sustained reductions in AMR will require stronger regulations, increased awareness of responsible antibiotic use, non-antimicrobial measures such as vaccination and infection control, and advanced waste and wastewater management practices. What remains unclear is how social determinants of health, anthropogenic activities, and the natural environment—which supports the health and sustainability of human, animal and plant populations—influence the evolution and spread of AMR.

The challenge lies in shifting away from addressing AMR within a single system and instead considering its evolution, spread and influence across all One Health systems, while developing evidence to assess risks across these interconnected systems. Achieving this will require integrating diverse datasets and establishing comparable measures that account for the different aetiologies [5]. Despite these challenges, the effort is worthwhile, as it will provide valuable insights into the impact of AMR both within individual systems and across the One Health framework. This evidence will support the community of practice—including researchers and policy makers—in making informed decisions about interventions to reduce the evolution and spread of AMR. These decisions are critical for mitigating the high burden of AMR and achieving sustained, equitable progress towards reducing this global threat.

## CRediT authorship contribution statement

**Yen Pham:** Writing – review & editing, Writing – original draft, Visualization, Validation, Software, Methodology, Investigation, Formal analysis, Data curation, Conceptualization. **Teresa M. Wozniak:** Writing – review & editing, Validation, Methodology, Conceptualization.

## Declaration of competing interest

The authors report no conflicts of interest in publishing this research manuscript.

## Data Availability

Data will be made available on request.
