# Supplementary material for: Systems thinking to understand the complexity of antimicrobial resistance across One Health: A systematic review of current approaches
Source: One Health. 2025 May 22;20:101081. doi: 10.1016/j.onehlt.2025.101081 (PMC12155812; doi:10.1016/j.onehlt.2025.101081)
Supplement: Supplementary material [file mmc1.docx]

# Appendix A. Supplementary materials

Table A.1. Factors influencing antimicrobial resistance identified from 21 selected studies

|  | Variables | | Relationship type* | References |
| --- | --- | --- | --- | --- |
| 1 | ABU in humans | AM residues in sewage and waste | + | [Frolova et al., 2020](#_ENREF_18) |
| 2 | ABU in humans | Effect of ABU in decreasing human infections | + | Authors** |
| 3 | ABU in humans | Effect of ABU in increasing AMR in humans | + | Authors |
| 4 | Advanced sewage and waste treatment | AM residues in sewage and waste | - | [Frolova et al., 2020](#_ENREF_18) |
| 5 | Agricultural profit | AMU in food animals | + | Brunton et al., 2019, Frolova et al., 2020, Sverdrup et al., 2020 |
| 6 | AM residues in sewage and waste | AM residues to the environment (water, soil) | + | [Frolova et al., 2020](#_ENREF_18) |
| 7 | AM residues to the environment (water, soil) | Evolution and prevalence of AMR in the environment | + | Cousins et al., 2023, Frolova et al., 2020 |
| 8 | AMU in food animals | AM residues in sewage and waste | + | [Frolova et al., 2020](#_ENREF_18) |
| 9 | AMU in food animals | Animal production rate | + | [Frolova et al., 2020](#_ENREF_18) |
| 10 | AMU in food animals | Effect of AMU in decreasing on-farm infections | + | Authors |
| 11 | AMU in food animals | Effect of AMU in increasing AMR in animals | + | Authors |
| 12 | Animal production rate | Agricultural profit | + | [Frolova et al., 2020](#_ENREF_18) |
| 13 | Animal vaccination | On-farm infectious disease burden | - | Brunton et al., 2019 |
| 14 | Appropriate AM prescribing and use | Effect of ABU in decreasing human infections | + | Authors |
| 15 | Appropriate AM prescribing and use | Effect of ABU in increasing AMR in humans | - | Authors |
| 16 | Appropriate AM prescribing and use | Effect of AMU in decreasing on-farm infections | + | Authors |
| 17 | Appropriate AM prescribing and use | Effect of AMU in increasing AMR in animals | - | Authors |
| 18 | Awareness of AMR | Appropriate AM prescribing and use | + | Aboah et al., 2023, Brunton et al., 2019 |
| 19 | Awareness of AMR | Infection prevention and control | + | Lambraki et al., 2022 |
| 20 | Effect of ABU in decreasing human infections | Human infectious disease burden | - | Authors |
| 21 | Effect of ABU in decreasing human infections | Need for new antibiotics | - | Authors |
| 22 | Effect of ABU in increasing AMR in humans | Evolution and prevalence of AMR in humans | + | Authors |
| 23 | Effect of ABU in increasing AMR in humans | Human infectious disease burden | + | Authors |
| 24 | Effect of ABU in increasing AMR in humans | Need for new antibiotics | + | Authors |
| 25 | Effect of AMU in decreasing on-farm infections | On-farm infectious disease burden | - | Authors |
| 26 | Effect of AMU in increasing AMR in animals | On-farm infectious disease burden | + | Authors |
| 27 | Effect of AMU in increasing AMR in animals | Evolution and prevalence of AMR in food animals | + | Authors |
| 28 | Enforcement and compliance of regulations | Advanced sewage and waste treatment | + | [Frolova et al., 2020](#_ENREF_18) *(Regulations on waste/wastewater management)* |
| 29 | Enforcement and compliance of regulations | Appropriate AM prescribing and use | + | Brunton et al., 2019, Cousins et al., 2023  *(Regulations related to antimicrobial stewardship, and compliance with treatment guidelines/AM prescribing)* |
| 30 | Enforcement and compliance of regulations | Infection prevention and control | + | Cousins et al., 2023, Homer et al., 2000  *(Regulations on infection prevention and control practices)* |
| 31 | Evolution and prevalence of AMR in food animals | Spread of AMR | + | Authors |
| 32 | Evolution and prevalence of AMR in humans | Morbidity and mortality | + | Homer et al., 2000, [Matthiessen et al., 2022](#_ENREF_30) |
| 33 | Evolution and prevalence of AMR in humans | Spread of AMR | + | Authors |
| 34 | Evolution and prevalence of AMR in the environment | Spread of AMR | + | Authors |
| 35 | Human infectious disease burden | ABU in humans | + | Brunton et al., 2019, Cousins et al., 2023 |
| 36 | Human vaccination | Human infectious disease burden | - | [Lambraki et al., 2023](#_ENREF_24) |
| 37 | Infection prevention and control | Human infectious disease burden | - | Homer et al., 2000, Lambraki et al., 2023, Lambraki et al., 2022 |
| 38 | Infection prevention and control | On-farm infectious disease burden | - | Homer et al., 2000, Lambraki et al., 2023 |
| 39 | Investment in new antibiotics | New antibiotics available | + | Homer et al., 2000 |
| 40 | Media attention | Awareness of AMR | + | Homer et al., 2000 |
| 41 | Media attention | Enforcement and compliance of regulations | + | Homer et al., 2000 |
| 42 | Morbidity and mortality | Media attention | + | Homer et al., 2000 |
| 43 | Need for new antibiotics | New antibiotics available | + | Frolova et al., 2020, Homer et al., 2000, Lambraki et al., 2023 |
| 44 | New antibiotics available | ABU in humans | + | Cousins et al., 2023, Homer et al., 2000, Lambraki et al., 2023, Lambraki et al., 2022 |
| 45 | On-farm infectious disease burden | AMU in food animals | + | Aboah et al., 2023, Brunton et al., 2019, Desbois et al., 2021 |
| 46 | Spread of AMR | Evolution and prevalence of AMR in food animals | + | Authors  *(Spread of AMR from humans to animals through airways; from the environment to animals through water or airways)* |
| 47 | Spread of AMR | Evolution and prevalence of AMR in humans | + | Authors  *(Spread of AMR from animals to humans through direct contact, consumption of contaminated products or airways; from the environment to humans through water or airways)* |
| 48 | Spread of AMR | Evolution and prevalence of AMR in the environment | + | Authors  *(Spread of AMR from humans and animals to the environment through waste or wastewater)* |

^*^ Relationship type refers to the positive (+) or negative (-) relationship from the variable in the first column to the variable in the second column within the same row but not vice versa unless stated otherwise.

^**^ Relationships between variables are based on findings from the selected studies and adjusted/specified by the authors to ensure logic coherence.

Abbreviations:

AM: Antimicrobials; ABU: Antibiotic use; AMR: Antimicrobial resistance; AMU: Antimicrobial use.

Table A.2. The structure of feedback loops presented in the causal loop diagram

| Name and structure of feedback loops |
| --- |
| ***Balancing feedback loops*** |
| **(B1)** |
| ABU in humans -> (+) Effect of ABU in decreasing human infections -> (-) Human infectious disease burden -> (+) ABU in humans |
| **(B2)** |
| AMU in food animals -> (+) Effect of AMU in decreasing on-farm infections -> (-) On-farm infectious disease burden -> (+) AMU in food animals |
| **(B3)** |
| ABU in humans -> (+) Effect of ABU in decreasing human infections -> (-) Need for new antibiotics -> (+) New antibiotics available -> (+) ABU in humans |
| **(B4)** |
| Appropriate AM prescribing and use -> (-) Effect of ABU in increasing AMR in humans -> (+) Evolution and prevalence of AMR in humans -> (+) Morbidity and mortality -> (+) Media attention -> (+) Awareness of AMR -> (+) Appropriate AM prescribing and use |
| **(B5)** |
| Appropriate AM prescribing and use -> (-) Effect of ABU in increasing AMR in humans -> (+) Evolution and prevalence of AMR in humans -> (+) Morbidity and mortality -> (+) Media attention -> (+) Enforcement and compliance of regulations -> (+) Appropriate AM prescribing and use |
| **(B6)** |
| ABU in humans -> (+) Effect of ABU in increasing AMR in humans -> (+) Evolution and prevalence of AMR in humans -> (+) Morbidity and mortality -> (+) Media attention -> (+) Awareness of AMR -> (+) Infection prevention and control -> (-) Human infectious disease burden -> (+) ABU in humans |
| **(B7)** |
| ABU in humans -> (+) Effect of ABU in increasing AMR in humans -> (+) Evolution and prevalence of AMR in humans -> (+) Morbidity and mortality -> (+) Media attention -> (+) Enforcement and compliance of regulations -> (+) Infection prevention and control -> (-) Human infectious disease burden -> (+) ABU in humans |
| **(B8)** |
| Appropriate AM prescribing and use -> (-) Effect of AMU in increasing AMR in animals -> (+) Evolution and prevalence of AMR in food animals -> (+) Spread of AMR -> (+) Evolution and prevalence of AMR in humans -> (+) Morbidity and mortality -> (+) Media attention -> (+) Awareness of AMR -> (+) Appropriate AM prescribing and use |
| **(B9)** |
| Appropriate AM prescribing and use -> (-) Effect of AMU in increasing AMR in animals -> (+) Evolution and prevalence of AMR in food animals -> (+) Spread of AMR -> (+) Evolution and prevalence of AMR in humans -> (+) Morbidity and mortality -> (+) Media attention -> (+) Enforcement and compliance of regulations -> (+) Appropriate AM prescribing and use |
| **(B10)** |
| ABU in humans -> (+) Effect of ABU in increasing AMR in humans -> (+) Evolution and prevalence of AMR in humans -> (+) Morbidity and mortality -> (+) Media attention -> (+) Awareness of AMR -> (+) Appropriate AM prescribing and use -> (+) Effect of ABU in decreasing human infections -> (-) Human infectious disease burden -> (+) ABU in humans |
| **(B11)** |
| ABU in humans -> (+) Effect of ABU in increasing AMR in humans -> (+) Evolution and prevalence of AMR in humans -> (+) Morbidity and mortality -> (+) Media attention -> (+) Enforcement and compliance of regulations -> (+) Appropriate AM prescribing and use -> (+) Effect of ABU in decreasing human infections -> (-) Human infectious disease burden -> (+) ABU in humans |
| **(B12)** |
| Advanced waste and wastewater treatment ->(-) AM residues in waste and wastewater ->(+) AM residues to the environment (water, soil) ->(+) Evolution and prevalence of AMR in the environment ->(+) Spread of AMR ->(+) Evolution and prevalence of AMR in humans ->(+) Morbidity and mortality ->(+) Media attention ->(+) Enforcement and compliance of regulations ->(+) Advanced waste and wastewater treatment |
| **(B13)** |
| ABU in humans ->(+) Effect of ABU in increasing AMR in humans ->(+) Evolution and prevalence of AMR in humans ->(+) Morbidity and mortality ->(+) Media attention ->(+) Awareness of AMR ->(+) Appropriate AM prescribing and use ->(+) Effect of ABU in decreasing human infections ->(-) Need for new antibiotics ->(+) New antibiotics available ->(+) ABU in humans |
| **(B14)** |
| ABU in humans ->(+) Effect of ABU in increasing AMR in humans ->(+) Evolution and prevalence of AMR in humans ->(+) Morbidity and mortality ->(+) Media attention ->(+) Enforcement and compliance of regulations ->(+) Appropriate AM prescribing and use ->(+) Effect of ABU in decreasing human infections ->(-) Need for new antibiotics ->(+) New antibiotics available ->(+) ABU in humans |
| **(B15)** |
| AMU in food animals ->(+) Effect of AMU in increasing AMR in animals ->(+) Evolution and prevalence of AMR in food animals ->(+) Spread of AMR ->(+) Evolution and prevalence of AMR in humans ->(+) Morbidity and mortality ->(+) Media attention ->(+) Awareness of AMR ->(+) Infection prevention and control ->(-) On-farm infectious disease burden ->(+) AMU in food animals |
| **(B16)** |
| AMU in food animals ->(+) Effect of AMU in increasing AMR in animals ->(+) Evolution and prevalence of AMR in food animals ->(+) Spread of AMR ->(+) Evolution and prevalence of AMR in humans ->(+) Morbidity and mortality ->(+) Media attention ->(+) Enforcement and compliance of regulations ->(+) Infection prevention and control ->(-) On-farm infectious disease burden ->(+) AMU in food animals |
| **(B17)** |
| ABU in humans ->(+) AM residues in waste and wastewater ->(+) AM residues to the environment (water, soil) ->(+) Evolution and prevalence of AMR in the environment ->(+) Spread of AMR ->(+) Evolution and prevalence of AMR in humans ->(+) Morbidity and mortality ->(+) Media attention ->(+) Awareness of AMR ->(+) Infection prevention and control ->(-) Human infectious disease burden ->(+) ABU in humans |
| **(B18)** |
| ABU in humans ->(+) AM residues in waste and wastewater ->(+) AM residues to the environment (water, soil) ->(+) Evolution and prevalence of AMR in the environment ->(+) Spread of AMR ->(+) Evolution and prevalence of AMR in humans ->(+) Morbidity and mortality ->(+) Media attention ->(+) Enforcement and compliance of regulations ->(+) Infection prevention and control ->(-) Human infectious disease burden ->(+) ABU in humans |
| **(B19)** |
| AM residues in waste and wastewater ->(+) AM residues to the environment (water, soil) ->(+) Evolution and prevalence of AMR in the environment ->(+) Spread of AMR ->(+) Evolution and prevalence of AMR in humans ->(+) Morbidity and mortality ->(+) Media attention ->(+) Awareness of AMR ->(+) Infection prevention and control ->(-) On-farm infectious disease burden ->(+) AMU in food animals ->(+) AM residues in waste and wastewater |
| **(B20)** |
| AM residues in waste and wastewater ->(+) AM residues to the environment (water, soil) ->(+) Evolution and prevalence of AMR in the environment ->(+) Spread of AMR ->(+) Evolution and prevalence of AMR in humans ->(+) Morbidity and mortality ->(+) Media attention ->(+) Enforcement and compliance of regulations ->(+) Infection prevention and control ->(-) On-farm infectious disease burden ->(+) AMU in food animals ->(+) AM residues in waste and wastewater |
| **(B21)** |
| AMU in food animals ->(+) Effect of AMU in increasing AMR in animals ->(+) Evolution and prevalence of AMR in food animals ->(+) Spread of AMR ->(+) Evolution and prevalence of AMR in humans ->(+) Morbidity and mortality ->(+) Media attention ->(+) Awareness of AMR ->(+) Appropriate AM prescribing and use ->(+) Effect of AMU in decreasing on-farm infections ->(-) On-farm infectious disease burden ->(+) AMU in food animals |
| **(B22)** |
| AMU in food animals ->(+) Effect of AMU in increasing AMR in animals ->(+) Evolution and prevalence of AMR in food animals ->(+) Spread of AMR ->(+) Evolution and prevalence of AMR in humans ->(+) Morbidity and mortality ->(+) Media attention ->(+) Enforcement and compliance of regulations ->(+) Appropriate AM prescribing and use ->(+) Effect of AMU in decreasing on-farm infections ->(-) On-farm infectious disease burden ->(+) AMU in food animals |
| **(B23)** |
| ABU in humans ->(+) AM residues in waste and wastewater ->(+) AM residues to the environment (water, soil) ->(+) Evolution and prevalence of AMR in the environment ->(+) Spread of AMR ->(+) Evolution and prevalence of AMR in humans ->(+) Morbidity and mortality ->(+) Media attention ->(+) Awareness of AMR ->(+) Appropriate AM prescribing and use ->(+) Effect of ABU in decreasing human infections ->(-) Human infectious disease burden ->(+) ABU in humans |
| **(B24)** |
| ABU in humans ->(+) AM residues in waste and wastewater ->(+) AM residues to the environment (water, soil) ->(+) Evolution and prevalence of AMR in the environment ->(+) Spread of AMR ->(+) Evolution and prevalence of AMR in humans ->(+) Morbidity and mortality ->(+) Media attention ->(+) Awareness of AMR ->(+) Appropriate AM prescribing and use ->(-) Effect of ABU in increasing AMR in humans ->(+) Human infectious disease burden ->(+) ABU in humans |
| **(B25)** |
| ABU in humans ->(+) AM residues in waste and wastewater ->(+) AM residues to the environment (water, soil) ->(+) Evolution and prevalence of AMR in the environment ->(+) Spread of AMR ->(+) Evolution and prevalence of AMR in humans ->(+) Morbidity and mortality ->(+) Media attention ->(+) Enforcement and compliance of regulations ->(+) Appropriate AM prescribing and use ->(+) Effect of ABU in decreasing human infections ->(-) Human infectious disease burden ->(+) ABU in humans |
| **(B26)** |
| ABU in humans ->(+) AM residues in waste and wastewater ->(+) AM residues to the environment (water, soil) ->(+) Evolution and prevalence of AMR in the environment ->(+) Spread of AMR ->(+) Evolution and prevalence of AMR in humans ->(+) Morbidity and mortality ->(+) Media attention ->(+) Enforcement and compliance of regulations ->(+) Appropriate AM prescribing and use ->(-) Effect of ABU in increasing AMR in humans ->(+) Human infectious disease burden ->(+) ABU in humans |
| **(B27)** |
| AM residues in waste and wastewater ->(+) AM residues to the environment (water, soil) ->(+) Evolution and prevalence of AMR in the environment ->(+) Spread of AMR ->(+) Evolution and prevalence of AMR in humans ->(+) Morbidity and mortality ->(+) Media attention ->(+) Awareness of AMR ->(+) Appropriate AM prescribing and use ->(+) Effect of AMU in decreasing on-farm infections ->(-) On-farm infectious disease burden ->(+) AMU in food animals ->(+) AM residues in waste and wastewater |
| **(B28)** |
| AM residues in waste and wastewater ->(+) AM residues to the environment (water, soil) ->(+) Evolution and prevalence of AMR in the environment ->(+) Spread of AMR ->(+) Evolution and prevalence of AMR in humans ->(+) Morbidity and mortality ->(+) Media attention ->(+) Awareness of AMR ->(+) Appropriate AM prescribing and use ->(-) Effect of AMU in increasing AMR in animals ->(+) On-farm infectious disease burden ->(+) AMU in food animals ->(+) AM residues in waste and wastewater |
| **(B29)** |
| AM residues in waste and wastewater ->(+) AM residues to the environment (water, soil) ->(+) Evolution and prevalence of AMR in the environment ->(+) Spread of AMR ->(+) Evolution and prevalence of AMR in humans ->(+) Morbidity and mortality ->(+) Media attention ->(+) Enforcement and compliance of regulations ->(+) Appropriate AM prescribing and use ->(+) Effect of AMU in decreasing on-farm infections ->(-) On-farm infectious disease burden ->(+) AMU in food animals ->(+) AM residues in waste and wastewater |
| **(B30)** |
| AM residues in waste and wastewater ->(+) AM residues to the environment (water, soil) ->(+) Evolution and prevalence of AMR in the environment ->(+) Spread of AMR ->(+) Evolution and prevalence of AMR in humans ->(+) Morbidity and mortality ->(+) Media attention ->(+) Enforcement and compliance of regulations ->(+) Appropriate AM prescribing and use ->(-) Effect of AMU in increasing AMR in animals ->(+) On-farm infectious disease burden ->(+) AMU in food animals ->(+) AM residues in waste and wastewater |
| **(B31)** |
| ABU in humans ->(+) AM residues in waste and wastewater ->(+) AM residues to the environment (water, soil) ->(+) Evolution and prevalence of AMR in the environment ->(+) Spread of AMR ->(+) Evolution and prevalence of AMR in humans ->(+) Morbidity and mortality ->(+) Media attention ->(+) Awareness of AMR ->(+) Appropriate AM prescribing and use ->(+) Effect of ABU in decreasing human infections ->(-) Need for new antibiotics ->(+) New antibiotics available ->(+) ABU in humans |
| **(B32)** |
| ABU in humans ->(+) AM residues in waste and wastewater ->(+) AM residues to the environment (water, soil) ->(+) Evolution and prevalence of AMR in the environment ->(+) Spread of AMR ->(+) Evolution and prevalence of AMR in humans ->(+) Morbidity and mortality ->(+) Media attention ->(+) Awareness of AMR ->(+) Appropriate AM prescribing and use ->(-) Effect of ABU in increasing AMR in humans ->(+) Need for new antibiotics ->(+) New antibiotics available ->(+) ABU in humans |
| **(B33)** |
| ABU in humans ->(+) AM residues in waste and wastewater ->(+) AM residues to the environment (water, soil) ->(+) Evolution and prevalence of AMR in the environment ->(+) Spread of AMR ->(+) Evolution and prevalence of AMR in humans ->(+) Morbidity and mortality ->(+) Media attention ->(+) Enforcement and compliance of regulations ->(+) Appropriate AM prescribing and use ->(+) Effect of ABU in decreasing human infections ->(-) Need for new antibiotics ->(+) New antibiotics available ->(+) ABU in humans |
| **(B34)** |
| ABU in humans ->(+) AM residues in waste and wastewater ->(+) AM residues to the environment (water, soil) ->(+) Evolution and prevalence of AMR in the environment ->(+) Spread of AMR ->(+) Evolution and prevalence of AMR in humans ->(+) Morbidity and mortality ->(+) Media attention ->(+) Enforcement and compliance of regulations ->(+) Appropriate AM prescribing and use ->(-) Effect of ABU in increasing AMR in humans ->(+) Need for new antibiotics ->(+) New antibiotics available ->(+) ABU in humans |
| ***Reinforcing feedback loops*** |
| **(R1)** |
| Evolution and prevalence of AMR in food animals ->(+) Spread of AMR ->(+) Evolution and prevalence of AMR in food animals |
| **(R2)** |
| Evolution and prevalence of AMR in humans ->(+) Spread of AMR ->(+) Evolution and prevalence of AMR in humans |
| **(R3)** |
| Evolution and prevalence of AMR in the environment ->(+) Spread of AMR ->(+) Evolution and prevalence of AMR in the environment |
| **(R4)** |
| ABU in humans ->(+) Effect of ABU in increasing AMR in humans ->(+) Human infectious disease burden ->(+) ABU in humans |
| **(R5)** |
| Agricultural profit ->(+) AMU in food animals ->(+) Animal production rate ->(+) Agricultural profit |
| **(R6)** |
| AMU in food animals ->(+) Effect of AMU in increasing AMR in animals ->(+) "On-farm infectious disease burden" ->(+) AMU in food animals |
| **(R7)** |
| ABU in humans ->(+) Effect of ABU in increasing AMR in humans ->(+) Need for new antibiotics ->(+) New antibiotics available ->(+) ABU in humans |

Abbreviations:

AM: Antimicrobials; AMU: Antimicrobial use; AMR: Antimicrobial resistance

# References

ABOAH, J., NGOM, B., EMES, E., FALL, A. G., SEYDI, M., FAYE, A. & DIONE, M. 2023. Mapping the effect of antimicrobial resistance in poultry production in Senegal: an integrated system dynamics and network analysis approach. *Frontiers in Veterinary Science,* 10.

BRUNTON, L. A., DESBOIS, A. P., GARZA, M., WIELAND, B., MOHAN, C. V., HäSLER, B., TAM, C. C., LE, P. N. T., PHUONG, N. T., VAN, P. T., NGUYEN-VIET, H., ELTHOLTH, M. M., PHAM, D. K., DUC, P. P., LINH, N. T., RICH, K. M., MATEUS, A. L. P., HOQUE, M. A., AHAD, A., KHAN, M. N. A., ADAMS, A. & GUITIAN, J. 2019. Identifying hotspots for antibiotic resistance emergence and selection, and elucidating pathways to human exposure: Application of a systems-thinking approach to aquaculture systems. *Science of the Total Environment,* 687**,** 1344-1356.

COUSINS, M., PARMLEY, E. J., GREER, A. L., NEITERMAN, E., LAMBRAKI, I. A., GRAELLS, T., LéGER, A., HENRIKSSON, P. J. G., TROELL, M., WERNLI, D., JORGENSEN, P. S., CARSON, C. A. & MAJOWICZ, S. E. 2023. Is scientific evidence enough? Using expert opinion to fill gaps in data in antimicrobial resistance research. *Plos One,* 18.

DESBOIS, A. P., GARZA, M., ELTHOLTH, M., HEGAZY, Y. M., MATEUS, A., ADAMS, A., LITTLE, D. C., HOG, E., MOHAN, C. V., ALI, S. E. & BRUNTON, L. A. 2021. Systems-thinking approach to identify and assess feasibility of potential interventions to reduce antibiotic use in tilapia farming in Egypt. *Aquaculture,* 540.

FROLOVA, L. L., SVERDRUP, A. E. & SVERDRUP, H. U. 2020. Using the Kaban Lakes Integrated Assessment Model for Investigating Potential Levels of Antibiotic Pollution of the Nizhniy Kaban and Sredniy Kaban Lakes. *Water, Air, and Soil Pollution,* 231.

HOMER, J., RITCHIE-DUNHAM, J., RABBINO, H., PUENTE, L. M., JORGENSEN, J. & HENDRICKS, K. 2000. Toward a dynamic theory of antibiotic resistance. *System Dynamics Review,* 16**,** 287-319.

LAMBRAKI, I. A., CHADAG, M. V., COUSINS, M., GRAELLS, T., LéGER, A., HENRIKSSON, P. J. G., TROELL, M. F., HARBARTH, S., WERNLI, D., JORGENSEN, P. S., CARSON, C. A., PARMLEY, E. J. & MAJOWICZ, S. E. 2023. Factors impacting antimicrobial resistance in the South East Asian food system and potential places to intervene: A participatory, one health study. *Frontiers in Microbiology,* 13.

LAMBRAKI, I. A., COUSINS, M., GRAELLS, T., LéGER, A., HENRIKSSON, P., HARBARTH, S., TROELL, M., WERNLI, D., JORGENSEN, P. S., DESBOIS, A. P., CARSON, C. A., PARMLEY, E. J. & MAJOWICZ, S. E. 2022. Factors influencing antimicrobial resistance in the European food system and potential leverage points for intervention: A participatory, One Health study. *Plos One,* 17.

SVERDRUP, H. U., FROLOVA, L. L. & SVERDRUP, A. E. 2020. Using a System Dynamics Model for Investigating Potential Levels of Antibiotics Pollution in the Volga River. *Water Air and Soil Pollution,* 231.
